# Nanosecond Electric Pulses as a Novel In Situ Vaccination Strategy for Cancer Treatment: Mechanisms, Challenges and Prospects

**DOI:** 10.3390/vaccines14070607

**Published:** 2026-07-10

**Authors:** Siqi Guo

**Affiliations:** Frank Reidy Research Center for Bioelectrics, Old Dominion University, Norfolk, VA 23508, USA; s2guo@odu.edu

**Keywords:** nanosecond electric pulses (nsEPs), cancer, ablation, in situ vaccination (ISV), abscopal effect, vaccine effect, immunogenic cell death (ICD), damage-associated molecular patterns (DAMPs), tumor microenvironment (TME), dendritic cell (DC) activation, T-cell immunity

## Abstract

Nanosecond electric pulses (nsEPs) are an emerging pulsed-power technology with unique bioelectric characteristics distinct from conventional long-pulse electroporation. As a tunable physical modality, nsEPs can modulate intracellular structures, membrane dynamics, and signaling pathways. Increasing evidence supports nsEPs as a promising non-thermal tumor ablation approach due to their high spatial precision, preservation of critical tissue structures, and minimal adverse effects. One of the most significant discoveries associated with nsEP tumor ablation is the induction of potent systemic antitumor immunity, particularly in situ vaccination (ISV) effects and, in some cases, abscopal effects against distant untreated tumors. Substantial evidence demonstrates that nsEPs can function as authentic immunogenic cell death (ICD) inducers by promoting the release of damage-associated molecular patterns (DAMPs), including calreticulin (CRT), ATP, and HMGB1. These events facilitate dendritic cell activation, antigen presentation, and the generation of long-term antitumor T-cell immunity. In addition to enhancing tumor immunogenicity, nsEPs profoundly remodel the tumor microenvironment (TME), including disruption of tumor vasculature, reduction in immunosuppressive cell populations, and alteration of stromal components. Emerging studies further suggest that nsEPs act as electric metabolic modulators capable of influencing mitochondrial function, calcium signaling, and metabolism-associated signaling pathways. Current evidence indicates that the immunological outcomes induced by nsEPs are highly dependent on pulse parameters, waveform characteristics, and tumor type. Despite its considerable therapeutic promise, the development of nsEP-induced ISV immunotherapy faces several important challenges, including standardization and optimization of pulse protocols, identification of critical molecular and cellular targets, and clarification of tumor- and cell-type-specific responses. Addressing these challenges through multidisciplinary collaboration and advanced technologies, including multi-omics, spatial analysis, and computational modeling, may accelerate the development of next-generation bioelectric immunotherapies for cancer treatment.

## 1. Introduction

Nanosecond electric pulses (nsEPs), also known as nanosecond pulsed electric fields (nsPEFs) or nano-pulse stimulation (NPS), represent an emerging class of sub-microsecond (1–999 ns), high-intensity (often >10 kV/cm) electrical stimuli capable of modulating cellular physiology through mechanisms distinct from traditional electroporation or pharmacological intervention [1,2,3]. Unlike microsecond–millisecond electric pulses, whose biological effects are dominated by charge accumulation across the plasma membrane, nsEPs can deliver energy on a timescale shorter than the membrane’s characteristic charging time (<100 ns) [3,4]. As a result, the electric field can penetrate beyond the plasma membrane and interact directly with intracellular structures. This unique ability has positioned nsEPs as a powerful platform for studying and modulating subcellular bioelectric organization, signaling dynamics, and stress responses [1,5,6,7].

A defining feature of nsEPs is the broad tunability of their physical characteristics. These pulses can vary in duration from a few to several hundred nanoseconds and can employ diverse waveforms, polarities, and temporal patterns [8,9,10]. Such versatility enables fine control over how electrical energy couples with biological targets, allowing investigators to access intracellular membranes, including mitochondria, endoplasmic reticulum, and nuclear envelopes—without the extensive permeabilization or thermal effects typically associated with longer pulses [1,4,11]. Consequently, nsEPs can influence mitochondrial membrane potential [12,13], intracellular calcium transients [5,14,15], redox status [16,17,18], and ion channel responses [19,20,21].

The mechanisms by which nsEPs act further distinguish them from conventional electroporation. Rather than relying on sustained pore formation in the plasma membrane, nsEPs operate primarily through rapid, non-thermal physical interactions with charged and polarizable biomolecules [1]. These interactions can reorganize membrane dipoles [22], perturb voltage-sensitive proteins [23], and trigger intracellular signaling cascades [7]. Importantly, these effects can occur without irreversible structural damage, enabling controlled modulation of physiological pathways such as apoptosis, differentiation, and metabolic regulation [18,24,25,26,27,28]. This intracellular reach and non-destructive mode of action represent a significant conceptual shift relative to classical electroporation, which is optimized for membrane permeabilization and drug/chemical delivery.

These unique attributes have stimulated growing interest in nsEPs for basic research and translational applications. In cancer biology, nsEPs have been explored for their ability to modulate mitochondrial function [29], disrupt bioelectric signaling [30], activate intracellular pathways [31,32], promote controlled cell death [33], and potentially modulate cell functions and behaviors of malignant cells [34]. Moreover, nsEPs provide a valuable tool for probing electrochemical gradients [35,36,37], ion channel behavior [20,23,38,39], and intracellular signaling architectures across diverse cell types [1,4,11,34]. Significantly, accumulating evidence suggests that nsEP tumor ablation can elicit potent antitumor immune responses, including immunogenic cell death (ICD), tumor microenvironment (TME) remodeling, in situ vaccination (ISV), and, in some cases, abscopal effects. These observations have generated increasing interest in nsEPs as a novel bioelectric immunotherapy platform. This review focuses on the immunological consequences of nsEP tumor ablation, the underlying mechanisms, current challenges, and prospects for clinical translation.

## 2. nsEP as an Emerging Technology for Cancer Ablation

nsEP technology has been studied and utilized in food industry, agriculture, basic sciences, and medicine [4]. Large efforts have been made to modulate cell and organ functions, deliver drugs, and treat various diseases including chronic wounds, infections, and cancer. Among all these applications, cancer ablation has been widely studied by numerous groups in various tumor models and clinical trials [40,41].

In preclinical settings, nsEP-based tumor ablation has demonstrated consistent efficacy across a range of cancer types. Extensive studies in murine melanoma models provided a benchmark for parameter optimization [42,43,44,45,46,47]. Early work using B16-F10 mouse melanoma exhibited that nsEP treatment (electric field strength 20–40 kV/cm, pulse duration 100–300 ns, and pulse number 100–2000) could result in high rates of complete long-term tumor regression [46,47]. Treated tumors are usually superficial (subcutaneous) and small (4–6 mm). Long-term tumor free survival can reach 90–100% [46,47]. Partial ablation is observed when field strength or pulse number is insufficient, often leading to tumor regrowth within 1–2 weeks. However, multiple treatments or increasing the dose (pulse number) of nsEPs can resolve the residual tumor tissue and improve complete regression and long-term survival [46,47,48].

nsEP technology is also widely studied in hepatocellular carcinoma (HCC) models. Early studies suggest that 100 ns is more efficient than 30 ns to ablate subcutaneous mouse Hepa1-6 tumors with complete regression 100% for pulse duration 100 ns vs. 40% for 30 ns [49]. Significantly, in a rat orthotopic N1S1 HCC model a high rate (91.3%) of complete regression was reached [50]. Noticeably, a needle array, which was used to deliver electric pulses, can be easily modified for clinical applications. In larger animal models such as rabbits, the translational feasibility of nsEP has been demonstrated by histological analysis, though long-term complete regression was not reported [51,52].

nsEP ablation has been explored in many other cancer models as well, including pancreatic [53,54], breast [55,56], colorectal [57], lung [58], and bladder [59]. Overall, nsEP treatment can prolong animal survival though complete regression and long-term survival rates are varied due to a number of impact factors such as pulse parameters, tumor type, and size. In general, the efficacy of nsEP ablation exhibits a dose-dependent response across all tumor models. Higher field strengths and pulse numbers increase the successful rate of complete response. Superficial tumors (e.g., melanoma, breast cancer) can achieve greater complete regression whereas deep-seated tumors (e.g., liver) face a great challenge to reach high efficacy. Additionally, large tumor size is a limiting factor for complete ablation. The complete regression and overall survival were significantly reduced when pancreatic cancer size increased from 4–7 mm to 8–11 mm [54].

From a safety perspective, nsEPs offer several distinct advantages. In contrast to radiofrequency or microwave ablation, nsEP-induced effects are largely non-thermal [42,47], minimizing collateral damage to adjacent healthy tissues. Histological analyses across multiple animal studies show sharply demarcated ablation zones with preservation of extracellular matrix architecture, major blood vessels, and connective tissue scaffolding [43,52]. This structural preservation facilitates tissue recovery and reduces the risk of complications such as hemorrhage or organ dysfunction.

Translation into clinical settings has further supported the favorable safety and efficacy profile of nsEP technology. Early human trials have primarily focused on liver [40] and skin cancers [60,61]. In patients with hepatocellular carcinoma, nsEP-based ablation has demonstrated effective local tumor control with minimal adverse effects, particularly in cases where tumors are located near critical structures that limit the use of thermal ablation [40]. Reported complications are mild and transient, including localized pain or edema, with low incidence of severe adverse events. Similarly, clinical studies in cutaneous malignancies such as basal cell carcinoma [60,61] show high lesion clearance rates, excellent cosmetic outcomes, and minimal scarring, reflecting the tissue-sparing nature of nsEP treatment.

Overall, nsEP represents a safe and effective emerging modality for cancer ablation. Its non-thermal mechanism and ability to preserve critical structures position it as a strong alternative or complement to existing ablation technologies. Noticeably, in addition to local tumor ablation, many studies have also demonstrated that nsEP treatment results in favorable immune outcomes in several cancer models.

## 3. Immunological Effects (In Situ Vaccination) Resulting from nsEP Tumor Ablation

Tumor-bearing animals treated with nsEP exhibit a range of immune outcomes, including reduction or elimination of distant lesions (abscopal effect) [43,51], inhibition or rejection of secondary tumor challenge (vaccine effect) [43,45,51,62] and enhanced T-cell infiltration and memory responses [43,51,57,58]. These observations suggest that nsEP tumor ablation not only induces local tumor destruction but also promotes systemic antitumor immunity. Importantly, unlike traditional tumor vaccines burdened by complex antigen pre-selection and time-consuming manufacturing constraints, this bioelectric platform harnesses the patient’s own tumor as a personalized antigen library. Rather than relying on ex vivo preparation, nsEP tumor ablation induces the in situ release of tumor-associated antigens alongside a synchronized cascade of damage-associated molecular patterns (DAMPs) from dying cells. This localized disruption subsequently primes a systemic host immune response, establishing nsEP as a non-pharmacological form of in situ vaccination (ISV).

### 3.1. Abscopal Effects

One of the most clinically relevant findings is the induction of systemic immunity following nsEP ablation. Using a two-tumor model, Nuccitelli et al. demonstrated that ablation of a primary B16 melanoma with nsEP significantly inhibited the growth of a second, untreated tumor compared to surgical removal of the primary lesion [48]. This result indicates that nsEP treatment can generate systemic antitumor activity beyond local tumor control. In a spontaneously metastatic 4T1 breast cancer model, Guo et al. showed that partial ablation of the primary tumor markedly reduced distant organ metastases [56]. Specifically, the incidence of metastasis decreased from 82% (9/11) in untreated animals to 14% (1/7) in nsEP-treated mice (*p* = 0.013), despite comparable primary tumor volumes between groups. Furthermore, residual tumors following nsEP treatment exhibited slower growth kinetics, and treated animals demonstrated either complete tumor clearance or prolonged survival.

However, the magnitude of the abscopal effect appears to be tumor-type-dependent. In pancreatic cancer models (Pan02), both Nuccitelli [63] and Guo [54] reported relatively weak systemic responses, with only ~10% (1/10) of animals rejecting distant tumors after primary tumor ablation. These findings suggest that intrinsic tumor properties, such as immunogenicity and microenvironmental factors, may limit the systemic efficacy of nsEP-induced immune responses. Even when tumor antigens are released following nsEP-mediated tumor ablation, efficient cross-presentation and subsequent cross-priming of tumor-specific T cells may not occur in the absence of sufficient danger signals or in the presence of an immunosuppressive TME. Consequently, combination strategies or immune adjuvants enhancing tumor immunogenicity and/or alleviating microenvironmental immunosuppression may be required to enhance these immunological effects in less responsive tumor types [63].

### 3.2. In Situ Vaccination Effects

In addition to abscopal responses, nsEP ablation can generate robust in situ vaccine effects, as evidenced by resistance to secondary tumor challenge. In a rat orthotopic hepatocellular carcinoma (N1S1) model, Chen et al. reported that 100% (21/21) of animals rendered tumor-free by nsEP treatment successfully rejected subsequent tumor rechallenge [45]. Similarly, in orthotopic 4T1 breast cancer models, Guo’s group demonstrated high protection rates, with 82% (22/27, *p* < 0.001) [64] to 100% (11/11, *p* < 0.001) [56] of tumor-free mice rejecting secondary tumor inoculation. These findings strongly support the development of durable, systemic antitumor immunity following nsEP ablation.

Importantly, treatment parameters significantly influence immune outcomes. In the Pan02 pancreatic cancer model, nsEP delivered at 100 ns, 50 kV/cm, and 800–1200 pulses resulted in a 75% (8/12) protection rate against tumor rechallenge [65], whereas a different parameter set (200 ns, 30 kV/cm, 600–1200 pulses) failed to induce protection (0%, 0/12) [54]. This highlights the critical role of pulse duration, field strength, and dose in shaping immunogenicity. In B16 melanoma, nsEP ablation produced moderate protection (33%, 6/18) against tumor rechallenge [61]. Even in cases where complete rejection was not achieved, residual tumors displayed slower growth, indicating partial immune control [54,56,65,66].

### 3.3. Ex Vivo Vaccination Effects

Beyond in situ vaccination, several studies have demonstrated that nsEP-treated tumor cells can function as a prophylactic vaccine. Immunization with nsEP-treated CT26 colorectal or EL4 lymphoma cells conferred protection rates of 78% (7/9) and 50% (3/6), respectively, against subsequent tumor challenge, whereas control animals showed no protection [67]. Similarly, vaccination with nsEP-treated MCA205 fibrosarcoma cells resulted in complete protection (100%, 6/6) [68]. In the 4T1 breast cancer model, immunization with nsEP-treated cells protected 50% (6/12, *p* < 0.05) of animals, while freeze–thaw tumor lysates failed to induce protection (0%, 0/12) [63]. These findings indicate that nsEP treatment preserves or enhances tumor immunogenicity compared to conventional cell death methods. Notably, the vaccine efficacy could be further improved by modulating oxidative stress during nsEP treatment (e.g., addition of Trolox or sodium pyruvate) [69], suggesting that treatment conditions influence antigen quality and immunogenic signaling.

### 3.4. Antitumor T-Cell Immunity

Evidence supporting the involvement of T cells in nsEP-induced antitumor immunity is emerging but remains variable across studies. Nuccitelli et al. reported increased CD4^+^ T-cell infiltration in both treated and distant melanoma tumors following nsEP ablation [43]. In a rat liver tumor model, elevated CD8^+^ T-cell levels were observed after treatment, and in the same study, depletion of CD8^+^ T cells abrogated the protective effect of vaccination with nsEP-treated MCA205 fibrosarcoma cells, demonstrating a functional requirement for CD8^+^ T cells in this context [68]. This CD8^+^ T-cell-dependent protection was confirmed in a human HPV16 transformed mouse (C3.43) tumor model treated with nsEP as well by the depletion of CD8^+^ T cells [62]. Further characterization by Guo’s group in orthotopic breast cancer models showed that animals cured by nsEP treatment exhibited significant increases in both CD4^+^ and CD8^+^ effector and central memory T cells [51,58]. These memory T cells displayed enhanced functional activity, including increased IFN-γ production and stronger responses upon tumor antigen rechallenge. Additionally, tissue-resident memory T cells (CD103^+^CD8^+^) were elevated as early as one week after nsEP ablation, indicating rapid initiation of local immune responses [58].

Despite these findings, it is important to note that direct mechanistic studies of T-cell dependency remain limited. While increased T-cell infiltration and memory formation are consistently observed in responsive models, comprehensive depletion or adoptive transfer experiments are still scarce. Therefore, although current evidence supports a central role for T cells—particularly CD8^+^ cytotoxic T cells—in mediating nsEP-induced antitumor immunity, further studies are needed to fully define their contributions across different tumor types. Since the abscopal effect (mediated by various anticancer therapies) had been demonstrated to rely specifically on CD8^+^ T-cell population (more precisely on CTL previously cross-primed), it is highly accepted that the same mechanism is operated in nsEP-induced antitumor immunity.

In summary, nsEP tumor ablation induces a spectrum of immunological effects ranging from local tumor control to systemic, long-lasting antitumor immunity. These effects include abscopal responses, ISV, and ex vivo vaccine potential, all of which are influenced by tumor type and treatment parameters. While T-cell-mediated immunity plays a key role, especially in models demonstrating durable protection, the extent and mechanisms of this involvement require further investigation to optimize nsEP as an immunotherapeutic strategy.

## 4. Mechanisms Behind nsEP-Induced Antitumor Immunity

Mechanisms underlying the strong abscopal and vaccine effects induced by nsEP ablation have been investigated by several groups. Accumulating evidence supports that nsEP functions as: (1) a novel physical inducer of immunogenic cell death (ICD); (2) a potent modifier of the tumor microenvironment (TME); and (3) a cellular metabolic reprogramming approach. Together, these mechanisms contribute to the generation of systemic antitumor immunity following local tumor ablation.

### 4.1. nsEPs as an Authentic Electric ICD Inducer

ICD is a regulated form of cell death capable of eliciting effective antitumor immune responses through activation of dendritic cells (DCs) and subsequent priming of T-cell immunity. Classical ICD inducers are anticancer modalities such as anthracyclines, oxaliplatin, radiotherapy, and photodynamic therapy [70]. Hallmarks of ICD comprise the release or exposure of damage-associated molecular patterns (DAMPs), including calreticulin (CRT), high mobility group protein B1 (HMGB1), ATP, and heat shock proteins, which promote antigen processing and immune activation [71,72,73,74,75].

#### 4.1.1. Release of ICD Surrogate Markers: CRT, ATP, and HMGB1

nsEP-induced DAMP release was first demonstrated by Nuccitelli et al., who reported CRT externalization in several cancer cell types, including rat (MCA-RH7777) hepatocellular carcinoma, mouse (SCC VII/SF) squamous carcinoma, and human (BxPC-3) pancreatic cancer cells [68]. Subsequent studies provided more comprehensive evidence supporting nsEP as a bona fide ICD inducer in multiple animal tumor models, based on the induction of canonical ICD hallmarks, DC activation, T-cell-mediated antitumor immunity, and successful in vivo vaccination assays. Nevertheless, whether nsEPs function as ICD inducers in human cancers remains to be established through clinical investigation.

Using a mouse orthotopic (4T1) breast cancer model, Guo’s group demonstrated that nsEPs fulfilled major criteria associated with authentic ICD induction. Specifically, nsEP exposure induced multiple DAMP signals, including CRT exposure, ATP secretion, and HMGB1 release [56]. Furthermore, DCs cocultured with nsEP-treated tumor cells exhibited increased expression of activation and costimulatory molecules, including CD40, CD80/86, and MHC-II, indicating enhanced antigen-presenting capacity [56,69]. Importantly, nsEP treatment also generated durable antitumor immune memory, characterized by elevated memory T-cell responses and protection against secondary tumor challenge [56,64]. In vaccination assays, considered the gold standard for ICD validation, animals immunized with nsEP-treated 4T1 cells developed significant protection against live tumor challenge [69].

#### 4.1.2. Function Validation by Vaccination Assay

**Figure 1:**
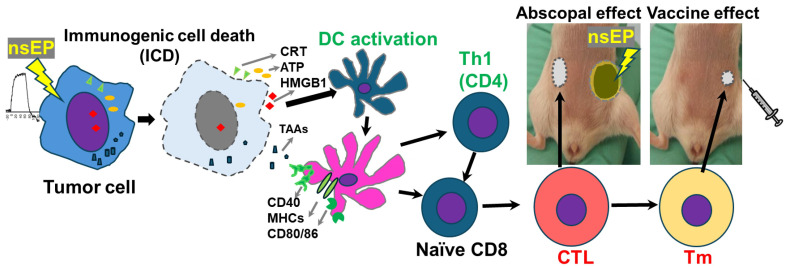
nsEPs function as an immunogenic cell death (ICD) inducer to elicit antitumor T-cell immunity. nsEP tumor ablation induces ICD in tumor (e.g., 4T1 mouse breast, SCC VII/SF mouse squamous, MCA-RH7777 rat hepatocellular, and BxPC-3 human pancreatic, EL4 human lymphoma, CT-26 human colorectal) cells, resulting in calreticulin (CRT) exposure, ATP secretion, and HMGB1 release. These damage-associated molecular patterns (DAMPs) promote dendritic cell (DC) activation, as indicated by upregulation of costimulatory molecules such as CD40, CD80/86, and MHC molecules. Simultaneously, dying tumor cells release tumor-associated antigens (TAAs), which are captured and processed by activated DCs. This process subsequently stimulates T-cell responses, leading to the generation of cytotoxic T lymphocytes (CTLs) that contribute to abscopal effects, as well as memory T cells (Tms) responsible for long-term in situ vaccination protection against tumor rechallenge. Note: While nsEP-induced ICD, DC activation, T-cell memory formation, ISV, and abscopal effects have been experimentally demonstrated, the proposed release of TAAs and their subsequent uptake and processing by DCs remain hypothetical and await direct experimental confirmation.

Independent studies by Rossi et al. further strengthened the concept of nsEP-induced ICD. In mouse EL4 lymphoma and CT26 colorectal cancer cells, nsEP treatment triggered canonical ICD-associated DAMP release, including CRT, ATP, and HMGB1 [61]. Similarly, Nuccitelli et al. demonstrated that vaccination with nsEP-treated MCA205 fibrosarcoma cells generated complete immune protection against tumor rechallenge [62]. Collectively, these studies establish nsEPs as a physical ICD inducer capable of generating antitumor immunity. A summary of schematic mechanisms, showing nsEPs as an ICD inducer to activate DCs and elicit T-cell immunity against distant lesions and second live tumor challenges, is shown in Figure 1.

#### 4.1.3. Pulse Parameter and Tumor-Type-Dependent Immunogenicity

However, the immunogenicity of nsEP-treated tumor cells appears to be highly tumor-type- and parameter-dependent. Vaccination with nsEP-treated MCA205 fibrosarcoma, CT26 colorectal, EL4 lymphoma, and 4T1 breast cancer cells resulted in tumor rejection rates of 100% (6/6), 78% (7/9), 50% (3/6), and 50% (6/12), respectively [67,68,69]. These differences suggest that intrinsic tumor immunogenicity, treatment conditions, and the magnitude of DAMP release collectively influence the strength of nsEP-induced immune responses. Indeed, subsequent studies indicate that nsEP does not universally induce ICD under all conditions, emphasizing the importance of parameter optimization for maximizing immunogenic outcomes. Recent report by Polajzer and Miklavcic [76] may explain this tumor-type- and nsEP-parameter-dependent immunogenicity difference. They found that electric pulse parameters (pulse type, duration, numbers/doses) influence the levels of DAMP release and which DAMP (CRT, ATP, HMGB1) was released.

### 4.2. nsEPs as a TME Modifier

In addition to inducing ICD, nsEPs have emerged as a potent modifier for the TME. TME consists of multiple cellular and non-cellular components, including endothelial cells, fibroblasts, pericytes, adipocytes, immune cells, extracellular matrix, cytokines, chemokines, and metabolites. Increasing evidence demonstrates that nsEP treatment can dramatically remodel many of these components, particularly by disrupting immunosuppressive networks within tumors.

#### 4.2.1. nsEPs Disrupt Tumor Vasculature

Both direct and indirect evidence show that nsEP treatment disrupts tumor vasculature. Initial study using transillumination imaging visualized rapid (within one day) and persistent (days to weeks) reduction in blood supply within nsEP-treated tumor [43]. Histological analysis further showed progressive decreases in intratumor vessel density from 7 to 17 days following nsEP exposure [27]. More recently, intravital multiphoton microscopy exhibited that nsEPs collapsed neovasculature and induced contraction of normal capillaries and larger vessels within 5–20 min [77].

Despite these observations, the precise mechanisms by which nsEPs rapidly alter tumor vasculature and maintain vascular disruption over prolonged periods remain poorly understood. Potential mechanisms, such as endothelial cell injury, vascular smooth muscle contraction, altered permeability, thrombosis, or vascular remodeling, should be further investigated.

#### 4.2.2. nsEPs Collapse Immunosuppressive Networks

Although early studies primarily focused on cytotoxic T-cell responses, more recent work demonstrates that nsEPs profoundly remodel the tumor immune microenvironment, particularly by dismantling immunosuppressive cellular networks. In the 4T1 breast cancer model, major immunosuppressive populations, including regulatory T cells (Tregs), tumor-associated macrophages (TAMs), and myeloid-derived suppressor cells (MDSCs), were significantly reduced following nsEP treatment [56,64].

Detailed temporal analysis revealed distinct response patterns among these cell populations [64]. Tregs and TAMs rapidly declined within 4 h after treatment, accompanied by increased cell death markers, suggesting direct cytotoxic effects of nsEPs. In contrast, MDSCs exhibited delayed reduction beginning approximately 2 days after treatment, despite minimal early cell death signals, implying indirect regulatory mechanisms. Importantly, nsEPs altered not only the abundance but also the function of these immunosuppressive cells. Treg suppressive activity decreased by approximately 50%, whereas TAMs upregulated MHC-II and CD86, consistent with polarization from immunosuppressive M2-like phenotypes toward immunostimulatory M1-like phenotypes [64]. An additionally noticeable observation was the selective sensitivity of Tregs, especially activated Tregs, to nsEP treatment. Conventional CD4^+^ and cytotoxic CD8^+^ T cells displayed minimal cytotoxicity following nsEP exposure, resulting in a continuing raised ratio of cytotoxic T cells to Tregs [64].

TME remodeling following nsEP treatment has been reported in other tumor models. In a mouse (Pan02) pancreatic cancer model, nsEP induced delayed MDSC reduction (at day 7), increased DC infiltration and activation, but also elevated Treg levels in the TME [54]. Single-cell RNA sequencing (scRNA-seq) further revealed substantial remodeling of myeloid populations, including increased monocyte/macrophage and DC abundance as well as elevated DC activation markers [78]. Another study confirmed macrophage expansion and further demonstrated selective reduction in granulocytic, but not monocytic MDSCs following nsEP treatment [79].

In a mouse (Hepa1-6) liver cancer model, nsEP treatment decreased Tregs (Foxp3^+^) and myeloid (CD11b^+^) cells while increasing macrophage (F4/80^+^) infiltration [80]. Another study demonstrated that nsEP-induced CD103^+^ DC increase and stimulation played a critical role in the CD8^+^ T-cell-mediated immune response in this model [81].

#### 4.2.3. nsEPs Alter Tumor Stroma

Limited evidence suggests that nsEPs may also remodel tumor stroma. Zhao et al. reported that nsEP treatment reduced extracellular matrix-associated proteins, including FAP-α and HABP1, in mouse pancreatic cancer models [79]. These stromal alterations may facilitate immune cell infiltration, improve drug penetration, and reduce physical barriers within tumors. However, stromal remodeling by nsEP remains largely underexplored and warrants further investigation.

**Figure 2:**
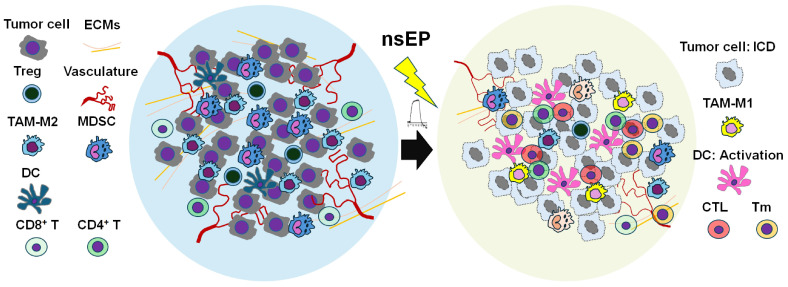
nsEP treatment remodels the tumor microenvironment (TME) to prime antitumor T-cell immunity. In addition to inducing immunogenic cell death (ICD) in tumor cells, nsEP treatment profoundly alters the TME, including: (a) disruption of tumor vasculature in mouse B16 melanoma and human U87 glioblastoma models; (b) collapse of immunosuppressive networks in mouse 4T1 breast, Hepa1-6 liver, and Pan02 pancreatic cancer models; and (c) stromal remodeling in the human Panc02 xenograft model. nsEPs markedly reduce major immunosuppressive cell populations, including regulatory T cells (Tregs), tumor-associated macrophages (TAMs), and myeloid-derived suppressor cells (MDSCs), while promoting TAM polarization from the immunosuppressive M2 phenotype toward the immunostimulatory M1 phenotype and enhancing dendritic cell (DC) activation. Together, these changes facilitate the generation of cytotoxic T lymphocytes (CTLs) and memory T cells (Tms), ultimately promoting systemic antitumor immunity and tumor elimination. Note: (1) The TME remodeling induced by nsEP has been demonstrated in selected animal models; however, its occurrence and characteristics in other tumor types require further experimental validation. (2) While the collapse of immunosuppressive networks is a recurring finding, the dynamics and composition of these changes may differ among tumor models.

The modification of TME by nsEPs contributing to T-cell immunity was summarized in Figure 2. Additionally, a comprehensive table (Table 1) summarizing tumor models, nsEP parameters, immune outcomes, and major mechanistic findings is included to facilitate cross-study comparisons.

### 4.3. nsEPs as an Electric Metabolic Modulator

It is well established that nsEPs can induce intracellular Ca^2+^ mobilization [5,14,15,82,83,84,85,86,87] and alter mitochondrial membrane potential [12,13,18,29,88]. Given the central role of Ca^2+^ signaling and mitochondria in regulating cellular metabolism, these findings strongly suggest that nsEPs can modulate metabolic processes. However, direct investigations of nsEP-induced metabolic reprogramming remain limited. Most previous studies have focused on either on cell excitation in excitable cells under physiological-level stimulation conditions [5,35,85,86,87] or on cytotoxicity in cancer cells using lethal or pharmacological pulse parameters [6,17,28,89].

Emerging evidence nevertheless supports the concept that nsEP functions as an electric metabolic modulator. Kamal et al. demonstrated that nsEPs could regulate both trans-plasma membrane electron transport and mitochondrial electron transport, as reflected by changes in oxygen consumption, in a dose-dependent manner [18], Importantly, these metabolic effects varied according to pulse source and waveform, suggesting that electrical parameters critically influence metabolic responses.

Several studies indicate that nsEPs alter metabolic signaling pathways associated with glycolysis and cellular stress adaptation. In human (MHCC97H) hepatocellular carcinoma cells, nsEP treatment downregulated glucose transporters GLUT1 and GLUT3 while activating JNK, AKT, and p38 signaling pathways [90]. Activation of MAPK signaling pathways (e.g., JNK, ERK, p38) following nsEP exposure has also been reported by multiple groups [31,91,92]. These pathways are closely linked to metabolic adaptation, mitochondrial function, stress response, and cell fate determination [93,94].

Accumulating evidence suggests that nsEP-induced metabolic modulation may contribute to broader biological outcomes, including cell differentiation [25,92], proliferation arrest [95,96], apoptosis [27,89], and immune activation [56,64]. However, whether immune effects are directly mediated by nsEP metabolic reprogramming remains unclear. Although cellular metabolism is tightly linked to immune cell function, direct experimental evidence connecting nsEP-induced metabolic alterations to antitumor immunity is currently lacking. Therefore, the potential contribution of metabolic reprogramming to nsEP-mediated immunomodulation should be regarded as a plausible but still hypothetical mechanism requiring further investigation.

## 5. Challenges

nsEPs are a pulsed-power technology capable of compressing electric energy into numerous waveform configurations, including monophasic or biphasic, square or no-square shapes, and varying rise/fall times, pulse durations, field strengths, frequencies, and pulse numbers. Unlike conventional vaccines or drugs, which are typically defined by a specific molecular structure and target, nsEP delivers complex electrical stimuli that simultaneously interact with multiple cellular and tissue components. This unique feature creates several major challenges for its development as an immunotherapeutic modality, including (1) standardization and optimization of nsEP protocol; (2) identification of relevant biological targets and mechanisms; and (3) understanding tumor- or cell-type-specific responses.

One of the most significant challenges is the large diversity of nsEP systems and pulse configurations developed by different research groups. Many studies employ distinct pulse generators and treatment conditions optimized for specific applications, making direct comparisons and reproducibility difficult. Even subtle changes in waveform characteristics can alter biological outcomes. For example, Dr. Xiao’s group demonstrated that minor variations in low-intensity post-pulse waveforms significantly changed cellular responses [97]. Similarly, as discussed above, ex vivo release of ICD-associated DAMPs was highly dependent on parameters [76]. ISV efficacy also appears pulse-parameter-dependent. In the same mouse (Pan02) pancreatic cancer model, one study using 200 ns pulses (200 ns, 30 kV/cm, 2 Hz, 600–1200 pulses) achieved no protection against tumor rechallenge (0/12) [54] whereas another study using 100 ns pulses (100 ns, 50 kV/cm, 3 Hz, 800–1200 pulses) reported 75% (8/12) protection [65]. These findings highlight the major challenge of optimizing and standardizing nsEP parameters to achieve consistent and robust immune responses. Given the nearly unlimited combinations of pulse parameters, systematic optimization remains a critical need in the field.

Another major challenge involves mechanistic studies. Traditional mechanistic research often focuses on a limited number of molecules or signaling pathways whereas nsEPs simultaneously affect numerous cellular components and processes, including membranes, ion channels, intracellular ions, cytoskeletal components, mitochondria, metabolites, and redox signaling. Theoretically, any charged atoms, molecules, and subcellular structures may be influenced to varying degrees by intensive electric pulses. Although membrane permeabilization [12,13,36,98,99], Ca^2+^ signaling [14,15,82,84,100,101], ion channel regulation [20,21], and ROS generation [16,17,69,102,103] have been relatively well studied, major critical molecules and pathways involved in ICD induction and ISV immunity remains poorly understood.

An additional challenge arises from the complexity of TME. nsEP ablation affects not only tumor cells but also stromal cells, immune cells, vasculature, extracellular matrix, and soluble signaling molecules. Because different tumor types possess highly heterogeneous cell TMEs, identifying which cell populations, molecule pathways, and tissue components are primarily responsible for generation of ISV immunity is extremely challenging. Furthermore, nsEP-induced TME remodeling likely involves dynamic and interconnected processes occurring at multiple spatial and temporal scales, further complicating mechanistic interpretation.

Despite these challenges, the complexity of nsEP-mediated bioelectric modulation also presents unique opportunities for developing more effective cancer immunotherapies. Several key considerations may help advance the field. First, pulse generators, waveform characteristics, and treatment parameters should be carefully defined and reported to improve reproducibility and cross-study comparisons. Second, systematic optimization of pulse conditions is essential for maximizing ICD induction and antitumor immune responses. Third, increased collaboration, resource sharing, and independent reproduction studies among research groups are needed to accelerate standardization and clinical translation. Fourth, elucidating the molecular mechanisms underlying nsEP-induced ICD and ISV may facilitate the development of rational combination therapies using immune modulators, metabolic regulators, or targeted drugs. Finally, deeper understanding of nsEP-TME interactions may identify novel strategies to overcome tumor immunoresistance and improve therapeutic efficacy.

Overall, although nsEP-ISV research faces substantial technical and mechanistic challenges, its ability to simultaneously modulate tumor cells, metabolism, and the immune microenvironment provides a promising foundation for the development of next-generation bioelectric cancer immunotherapies.

## 6. Prospects

In contrast to highly effective prophylactic vaccines against infectious diseases (e.g., polio, measles, hepatitis B, pneumococcus, meningococcus), therapeutic cancer vaccines so far have limited efficacy or minimal clinical benefit in many clinical trials [104]. One major obstacle facing classical cancer vaccines is the highly immunosuppressive TME, which restricts effective immune activation and T-cell function. In this regard, ISV strategies possess a unique advantage because they can simultaneously induce antigen release and remodel the local TME. Notably, nsEPs not only induce ICD in tumor cells but also disrupt immunosuppressive networks within the TME. The strong immune outcomes observed after a single nsEP treatment in multiple tumor models, including ISV protection and abscopal effects [48,50,56,63,64], highlight its considerable potential as a novel cancer immunotherapy platform.

Nevertheless, serious questions and challenges remain due to the unique characteristics of this emerging bioelectric technology. Addressing these issues will require well-designed mechanistic and translational studies aimed at optimizing nsEP protocols for immune enhancement, elucidating underlying molecular and cellular mechanisms, and validating therapeutic efficacy in clinical trials.

Given the clinical significance of nsEP-induced immune responses, future research will focus on optimizing pulse parameters for specific cancer types and therapeutic goals. It remains unclear whether previously reported low or absent immune protection in certain tumor models reflects intrinsic tumor resistance or suboptimal pulse configurations. Therefore, systematic evaluation of pulse duration, electric field strength, pulse number, waveform, and frequency will be essential to maximize immunogenicity and therapeutic benefit. Several studies have already demonstrated that nsEP-induced immune responses can be further enhanced through combination with immune stimulators or adjuvants, including Toll-like receptor (TLR) agonist [63,80]. Accordingly, rational combination strategies integrating nsEP with immune modulators, checkpoint inhibitors, metabolic therapies, or cytokine-based approaches may provide improved efficacy, particularly for poorly immunogenic and immunologically ‘cold’ tumors.

As discussed above, many questions remain regarding the precise mechanisms underlying nsEP-induced ISV immunity, including the major cell populations, signaling pathways, and molecular mediators involved. Clarifying these mechanisms will require multidisciplinary collaboration integrating pulsed-power engineering, electrophysiology, cancer biology, immunology, metabolism, bioinformatics, and systems biology. Emerging technologies such as scRNA-seq, spatial transcriptomics, spatial metabolomics, multiplex imaging, and AI-assisted computational modeling are expected to provide valuable insight into the dynamic interactions between tumor cells, immune cells, stromal components, and electrical stimulation. These approaches may help define the temporal evolution of immune responses following nsEP treatment, identify predictive biomarkers, and uncover novel signaling pathways involved in nsEP-mediated antitumor immunity. In particular, the roles of nsEP-regulated calcium signaling, mitochondrial dysfunction, ROS, MAPK pathways, and metabolic reprogramming warrant further investigation.

To date, most studies examining nsEP-induced immunity have focused primarily on tumor ablation and enhancement of tumor immunogenicity. In contrast, the direct modulation of immune cells by nsEP has not been systematically explored. Although increased DC activation following nsEP treatment has been reported in several tumor models [54,56,78,81], it remains difficult to distinguish whether this activation results directly from electrical stimulation or indirectly from DAMP release by dying tumor cells. Interestingly, one study reported that nsEP could directly activate DCs ex vivo [56], suggesting that nsEP itself may function as an electric immune adjuvant. Similarly, whether the observed alterations in Tregs, MDSCs, and TAMs result from direct nsEP exposure or occur secondary to TME remodeling remains unclear.

Given that nsEPs can modulate intracellular Ca^2+^ signaling, redox status, mitochondrial function, membrane potential, and MAPK activation, all of which are fundamental regulators of immune cell metabolism and function, it is highly plausible that nsEP directly influences immune cell behavior. Therefore, systematic investigation of nsEP-mediated immune cell modulation represents an important future research direction. Such studies may not only improve understanding of nsEP-induced ISV immunity but also facilitate the development of next-generation bioelectric immunotherapies capable of precisely modulating both tumor cells and immune cells for improved cancer control.

## 7. Limitations

Several limitations should be considered when interpreting the findings summarized in this review.

First, although substantial evidence supports the ability of nsEPs to induce ICD, remodel the TME, and generate ISV effects, some findings remain inconsistent across studies. Variations in tumor models, pulse parameters, treatment protocols, and experimental endpoints have occasionally produced divergent immune outcomes. For example, the magnitude of vaccine protection, abscopal effects, and immune cell responses differs among tumor types and treatment conditions (Table 1). These discrepancies highlight the need for cautious interpretation and systematic validation across independent laboratories.

Second, reproducibility remains a significant challenge in the field. Unlike conventional drugs, nsEPs are defined by multiple physical parameters, including pulse duration, electric field strength, waveform, polarity, frequency, and pulse number. Even subtle differences in pulse delivery systems or treatment conditions may alter biological responses. Consequently, direct comparisons between studies are often difficult, underscoring the need for standardized reporting guidelines, protocol harmonization, and multicenter validation studies.

Third, this review primarily focuses on nsEP-mediated remodeling of the tumor microenvironment as a mechanistic basis for antitumor immunity. Although systemic immune outcomes, including T-cell memory formation, vaccine effects, and abscopal responses, are discussed, a comprehensive analysis of systemic immunology was beyond the scope of this review. Future studies integrating both local TME remodeling and systemic immune responses will be necessary to establish a more complete understanding of nsEP-induced antitumor immunity.

Fourth, despite growing evidence linking nsEP treatment to ICD induction, immune activation, and TME remodeling, the underlying molecular mechanisms remain incompletely understood. Direct causal pathways connecting bioelectric perturbation, ion fluxes, mitochondrial dysfunction, metabolic reprogramming, and immune responses have not been fully elucidated. Most available studies are descriptive, and mechanistic investigations identifying key signaling networks and molecular mediators remain limited.

Finally, clinical evidence supporting nsEP-induced immune responses is currently scarce. While encouraging preclinical data have been generated in multiple animal tumor models (Table 1), clinical studies [40,60,61] have primarily focused on safety and local tumor control rather than immune outcomes. Important translational questions remain regarding treatment optimization, patient selection, biomarker development, long-term immune monitoring, and integration with existing immunotherapies. Therefore, the clinical relevance and therapeutic potential of nsEP-induced ISV and TME remodeling require further validation in well-designed human studies.

## 8. Conclusions

Nanosecond electric pulses (nsEPs) represent a unique bioelectric modality that combines precise non-thermal tumor ablation with the capacity to induce systemic antitumor immunity. Accumulating evidence demonstrates that nsEPs can function as authentic ICD inducers, remodel the TME, and modulate cellular metabolism, collectively contributing to ISV effects and, in some cases, abscopal effects. Compared with conventional cancer therapies, nsEPs offer distinct advantages including intracellular targeting, preservation of critical tissue structures, and minimal side effects. However, the immunological outcomes of nsEP treatment remain highly dependent on pulse parameters and tumor type, highlighting the need for protocol standardization and mechanistic clarification. Future studies should define the molecular pathways linking bioelectric perturbation to immune activation and determine how nsEP-induced antitumor immunity can be optimized across diverse tumor settings. Combination strategies that enhance tumor immunogenicity and alleviate immunosuppression warrant further investigation to maximize the therapeutic potential of nsEP-induced immune responses. Future multidisciplinary studies integrating pulsed-power engineering, immunology, metabolism, and advanced multi-omics technologies are expected to accelerate the development of nsEP-based bioelectric immunotherapies for effective cancer management.

## Figures and Tables

**Table 1:** Summary of nsEP-induced immune outcomes and key mechanistic findings in various cancer models.

Cancer Types	nsEP Parameters	Immune Outcomes	Key Mechanistic Findings
Breast cancer (mouse 4T1), orthotopic.	100 ns, 50 kV/cm, 1–3 Hz, 600–1000 pulses [56,64].	ISV: Protection from second challenge, 82% vs. 0% (22/27 vs. 0/14, *p* < 0.001), 100% vs. 0% (11/11 vs. 0/11, *p* < 0.001). Abscopal effect: Reduction in distant organ metastases, 14% vs. 82% (1/7 vs. 9/11, *p* = 0.013). T-cell immunity: Increase in both effector and central CD4^+^ and CD8^+^ T cells, antigen-specific IFN-γ release.	Induction of ICD: Release of CRT, ATP, and HMGB1. DC activation in vitro: Upregulation of CD40, CD86, MHC-II. Depletion of immunosuppressive cells (Tregs, TAMs, MDSCs) in the TME. Induction of cell death for Tregs and TAMs whereas minimal impact on conventional CD4^+^ and CD8^+^ T cells. Promotion of tissue memory T cells.
Melanoma (mouse B16), subcutaneous.	200 ns, 25 kV/cm, 2 Hz, 750 pulses [66].	ISV: Protection from second challenge, 33% vs. 0% (6/18 vs. 0/20, *p* < 0.05).	
Pancreatic (mouse Pan02), subcutaneous.	100 ns, 50 kV/cm, 3 Hz, 800–1200 pulses [65].	ISV: Protection from second challenge, 75% vs. 0/12 (8/12 vs. 0/12, *p* = 0.001).	
	200 ns, 30 kV/cm, 2 Hz, 600–1200 pulses [54].	ISV: No protection from second challenge, 0% (0/12). Abscopal effect: Rejection of distant lesion, 10% (1/10). T-cell immunity: No increase in effector and central CD4^+^ and CD8^+^ T cells.	DC activation in the tumor: Upregulation of CD40, CD86, MHC-II. Inconsistent alteration in immunosuppressive cells in the TME: Increase in Tregs at day 2 but decrease at day 7, and no change in MDSCs at day 2 but decrease at day 7.
Pancreatic (mouse Pan02), intradermal.	180 mJ/mm^3^ or 360 mJ/mm^3^ (no detailed parameters are disclosed) [63].	ISV: Only slight inhibition of second challenge tumor. Abscopal effect: Rejection of distant lesion, 10% (1/10).	
Hepatocellular (mouse Hepa1-6), subcutaneous.	100 ns, 20 kV/cm, 1 Hz, 100 pulses [80].		Induction of ICD: Release of CRT, ATP, and HMGB1. DC activation in the tumor: Upregulation of CD80/86. Decrease in Tregs and CD11b^+^ myeloid cells but increase in F4/80^+^ macrophages.
Hepatocellular (rat N1S1), orthotopic.	100 ns, 50 kV/cm, 1 Hz, 1000 pulses [50].	ISV: Protection from second challenge, 100% vs. 0% (21/21 vs. 0/23, *p* < 0.001). T-cell immunity: Presence of granzyme B-secreting T cells.	
Hepatocellular (rat McA-RH7777), orthotopic.	100 ns, 50 kV/cm, 400 pulses [68].	ISV: Reduction (4/6) or rejection (2/6) of second tumor challenge.	CD8^+^ T-cell dependency: Histology showing CD8^+^ T-cell increase in the tumor. Depletion of CD8^+^ T cells reverses the growth inhibition of the second tumor.
Fibrosarcoma (mouse MCA205), subcutaneous.	100 ns, 50 kV/cm, 500 pulses [68].	In vivo vaccination protection: Protection from live tumor challenge, 100% vs. 0% (6/6 vs. 0/6, *p* < 0.001).	CD8^+^ T-cell dependency: Depletion of CD8^+^ T cells cancels the vaccination protection.
Breast cancer (mouse 4T1), orthotopic.	60 ns, 50 kV/cm, 1 Hz, 120 pulses [69].	In vivo vaccination protection: Protection from live tumor challenge, 50% vs. 0% (6/12 vs. 0/12, *p* < 0.05).	DC activation in vitro: Upregulation of CD40, CD80/86, MHC-I/II.
Lymphoma (mouse EL-4), subcutaneous.	200 ns, 7 kV/cm, 10 Hz, 200 pulses [67].	In vivo vaccination protection: Protection from live tumor challenge, 50% vs. 11% (3/6 vs. 1/9).	Induction of ICD: Release of CRT, ATP, and HMGB1.
Colorectal (mousCT-26), subcutaneous.	200 ns, 7 kV/cm, 10 Hz, 600 pulses [67].	In vivo vaccination protection: Protection from live tumor challenge, 78% vs. 0% (7/9 vs. 0/10).	Induction of ICD: Release of CRT, ATP, and HMGB1.

## Data Availability

No new data was created. All data cited came from published literature.

## Abbreviations

The following abbreviations are used in this manuscript:

ATP	Adenosine triphosphate
CRT	Calreticulin
CTL	Cytotoxic T cells
DAMPs	Damage-associated molecular patterns
DCs	Dendritic cells
HCC	Hepatocellular cancer cells
HMGB1	High mobility group protein B1
ICD	Immunogenic cell death
ISV	In situ vaccination
MDSCs	Myeloid-derived suppressor cells
MHC	Major histocompatibility complex
nsEPs/PEFs	Nanosecond electric pulses/pulsed electric fields
NPS	Nano-pulse stimulation
ROS	Reactive oxygen species
Sc-RNAseq	Single-cell RNA sequencing
TAAs	Tumor-associated antigens
TAMs	Tumor-associated macrophages
TLR	Toll-like receptor
Tms	Memory T cells
TME	Tumor microenvironment
Tregs	Regulatory T cells
